# 

**Published:** 1859-06

**Authors:** 


### INDEX TO VOLUME FIFTEENTH.

For original communications, look for the names of the authors as well as the title of the subjects.

For selected and editorial articles, look for the titles of the subjects only.

For notices of books, look for names of authors.

### ILLUSTRATIONS.

Lente's Modification of the N. Y. Hospital Splint for Fractured Thigh....19, 20
Vertical Section of Female Pelvis. Dr. Peaslee's Lecture, No. I........... 582
Dr. H. G. Davis' Splint for Hip Disease (Frontispiece)..................... 657
Vaginal Syringe—Dr. Peaslee's Lecture, No. II.............................. 670
Dr. Bauer's "Wire Breeches" for Hip Disease................................ 715
Photographs of Case of Hip Disease. Dr. Sayre.....................724, 725

Abortion of Triplets—Case of.................................................. 313
Academy of Medicine.........................................................354, 644
Acetic Acid in Erysipelas...................................................... 623
Alum Pastilles................................................................... 694
American Medical Association—Twelfth Annual Meeting................. 41
Aneurism—Case of................................................................ 188
Aneurism—Case of, by Digital Compression.................................. 180
Anæsthetics on Horses—Action of............................................. 811
Anæsthesia during Sleep......................................................... 789
Annual Meeting of the N. Y. State Medical Society........................ 632
Arsenious Acid in Apoplectic Congestions................................... 612
Arsenical Paper-Hangings (A. S. Taylor, M.D.)............................. 37
Asthma—(Hyde Latter)............................................................ 358
Asphyxia Neonatorum.............................................................. 616
Asthma................................................................................ 709
Belladonna in Preventing Secretion of Milk.................................. 426
Belladonna as an Anti-Galactic...............................................620, 790
Bird on Urinary Deposits......................................................... 424
Blood as Food.....................................................................728, 872
Bloodletting in Inflammation................................................... 695
Braithwaite's Retrospect—Notice of.......................................... 805
British and Foreign Medico-Chirurgical Review—Notice of................ 806
British Army and Navy Medical Service...................................... 182
Bronchitis............................................................................ 619
Bronchocele......................................................................... 620
Brown-Séquard on Contraction of Excretory Ducts in Birds................ 174
Brown-Séquard on Restoring Individuals to Life Dying of Disease......... 105
Buffalo Medical Association—Proceedings of.........76, 291, 416, 487, 540, 649
Butler (Dr. Wm. H.)—Case of Phymosis....................................... 421

Cancer of the Breast—Spontaneous Cure of .......... 693
Cardiac Hypertrophy and Dilatation (Flint).......... 493
Caustic Potassa—Researches on the Action of .......... 604
Chloride of Zinc as a Caustic .......... 692
Chloro-Anæmia treated with St. Ignatius Bean .......... 601
Chloroform and Cod-Liver Oil in Diphtheritis .......... 618
Cholera Infantum Cured by a Poisonous Dose of Fowler's Solution .......... 626
Chronic Rheumatism .......... 707
Clerical Quackery, by O. C. Gibbs, M.D.......... 418
Color Blindness .......... 570
Coloring the Lips after Cheiloplasty .......... 373
Congestive Chills—New Treatment for .......... 874
Consumption—A New Remedy for .......... 875
Convention for Revising the U. S. Pharmacopœia.......... 189
Coryza Maligna .......... 862
Criminal Abortions in America (Storer)—Notice of.......... 805
Croup—Treatment of. .......... 878
Cystic Degeneration of Kidney.......... 809
Dalton, Jr. (Dr. John C.)—Lectures on the Physiology of the Circulation..321, 585
449, 513, 737, 830
Damerinville's (L.) Trans. of Brown-Séquard's Article on Contraction of Excretory Ducts .......... 174
Davis (Dr. H. G.) on Mechanical Means in the Treatment of Morbus Coxarius 678
Dewees (Dr. H. P.) on Pyæmia .......... 817
Dickson's Elements of Medicine .......... 175
Diphtheria ..........610, 630, 699, 796
Diseases of the Ear—Toynbee—Notice of .......... 803
Diseases of the Tropics .......... 588
Displacements of the Uterus—Lectures on ..........577, 668, 758, 852
Dowler, Dr. Bennett .......... 187
Dragées of Iron, Manna, and Bismuth.......... 690
Durkee on Gonorrhœa and Syphilis.......... 356
Dysentery ..........700, 784
Editorial and Miscellaneous ..........652, 726, 814, 895
Edmunds (Dr. J. J.)—Letter from.......... 444
Edmunds (Dr. J. J.)—Case of Gunshot Wound through the Abdomen .......... 149
Elephantiasis .......... 786
Electricity—Application of in Medical, Surgical, and Obstetric Practice, by Theophilus Mack, M.D.......... 7
Electricity—Is it an Anæsthetic ?.......... 427
Elwell on Malpractice and Medical Evidence—Review of .......... 801
Endermic Medication.......... 702
Epilepsy—Treatment of .......... 710
Erysipelas—Treatment of in Limbs by Elevation (Henry) .......... 247
Ether as an Anæsthetic.......... 571
Excised Knees.......... 113
Extra-Uterine Pregnancy .......... 701
Extra-Uterine Gestation.......... 812
Fifteenth Volume—Introductory to.......... 40

Flint on Diseases of the Heart.......................................... 319
Flint (Dr. Austin) on Case of E. A. Groux.................................... 1
Flint's Report of Cases at the New Orleans Charity Hospital ......129, 193, 257
Fracture of Anatomical Neck of Humerus, etc., by Frederic Lente, M.D..... 65
Fracture of the Skull—Case of.......................................... 311
Fracture of Thigh—N. Y. Hospital Apparatus for, by Frederic Lente, M.D. 15
Friction Sound from Thickening of the Peritoneum.......................... 573
Frick (Dr. Charles)—Death of ........................................ 732
Fungoid Growth of the Gums and Jaws.................................... 881
Gastrotomy................................................................ 626
Gelsemin ......................................................617, 788
Gibbs' (Dr. O. C.) Monthly Summary of Medical Journalism, 630, 712, 784, 872
Gibbs (Dr. O. C.)—Letter from ........................................... 506
Gibbs (Dr. O. C.) on Clerical Quackery.................................... 418
Glucosuria in Paludal Fever.............................................. 694
Goitre—Cause of ......................................................... 874
Gonorrhœa—Treatment of .................................................. 795
Gray's Descriptive and Surgical Anatomy ................................. 422
Graham (Dr. J. N.)—Perchloride of Iron Injections for Uterine Polypus .... 599
Gross' System of Surgery.................................................. 317
Gross' Appointment to Howard Hospital ................................... 319
Gunshot Wound through the Abdomen—Case of, by Dr. J. J. Edmunds .... 149
Hæmaturia................................................621, 787
Hamilton's (Dr. F. H.) Treatise on Fractures and Dislocations—Review of.. 797
Headland on the Action of Medicines...................................... 492
Hepatic Abscess........................................................... 786
Hernia—Radical Cure of .................................................. 704
Hernia, Strangulated—Treated with Opium ................................. 880
Holcomb (Dr.)—Letter from ............................................... 727
Hospitals, etc, of New York........................248, 307, 376, 428, 500
Hospital Statistics ....................................................... 445
Hydrophobia—Case of Successfully Treated with Calomel ................. 557
Hydrocephalus ............................................................ 787
Hydrochloric Acid in Small-Pox............................................ 735
Hydrophobia............................................................... 699
Hypophosphites in Phthisis ............................................... 626
Hypophosphites in Spurious Hydrocephalus ................................ 879
Indian Corn an Anti-Periodic.............................................. 788
Indolent Ulcers—Treatment of ............................................ 623
Induction of Premature Labor............................................. 705
Inflammation—Treatment of by Digital Compression ....................... 367
Influence of the Mother's Mind on the Fœtus............................... 706
In-growing Nail ......................................................... 615
Injuries of the Skull .................................................... 793
Intermittents—Treatment of............................................... 623
Intestinal Infusoria in Man (Malmstem).................................... 498
Inversion of the Uterus..........................................621, 875
Iodide of Potassium in Diseases of the Brain in Children.................. 626
Iodide of Potassium (Power of) in Expediting Mercurial Salivation ......... 586

Iodide of Potassium in Neuralgia........................ 881
Iodine Injections in Lymphatic Tumors........................ 623
Journal de la Physiologie........................ 300
Kelly (Dr. Bernard) on Coryza Maligna........................ 862
Kelly (Dr. Bernard) on Iodide of Potassium in Mercurial Salivation........ 586
Langenbeck (Prof. B.) on Plastic Operations........................ 657
Lectures on Displacements of the Uterus....................577, 668, 758, 852
Lectures on the Physiology of the Circulation .......321, 385, 449, 513, 737, 830
Lente (Dr. Frederic)—Modification of N. Y. Hospital Apparatus for Fracture of Thigh........................ 15
Lente (Dr. Frederic)—Fracture of Anatomical Neck of Humerus.......... 65
Letter from Buffalo........................ 252
Lithotrity—Different Modes of Performing in the English Hospitals........ 375
Light on Vegetable and Animal Fæcula........................ 602
Long Island College Hospital........................ 509
Lumbar Abscess........................ 768
Lupulin in Delirium Tremens........................ 617
Mack (Dr. Theophilus)—Medical Application of Electricity............... 7
Mack's (Dr. T.) Monthly Review of Parisian Medicine and Surgery....24, 72, 151 274, 533
Malarial Fever........................ 876
Malgaigne on Fractures........................ 171
Meconium and Vernix Caseosa—Identity of........................ 306
Medical Education in New Orleans........................ 254
Medical Journals—Suspension of........................ 126
Medical Colleges—Statistics of........................ 191
Medical Schools—Opening of........................ 382
Medical Astronomer........................ 650
Medico-Legal Treatise on Malpractice—Review of........................ 891
Medical Science among some East India Nations........................ 684
Meigs on Diseases of Women........................ 191
Melanotœ Cancer—Diagnosis of by the Urine (Dr. Eiselt)................ 128
Mercury—Action of on Secretion of Bile............................320, 369
Mercurial Salivation Expedited by Iodide of Potassium.................. 586
Morbus Coxarius........................ 712
Morbus Coxarius—Treated by Mechanical Means........................ 678
Movable Bodies in Synovial Sac........................ 316
Navy Medical Board........................ 127
Newman (Dr. J. M.)—Removal from Buffalo........................ 508
New Medical Journal........................ 512
New Orleans Charity Hospital—Report of Cases, by Prof. A. Flint..129, 193, 257
New Sydenham Society........................ 447
N. Y. Pathological Society—Proceedings of..85, 156, 211, 280, 342, 404, 467, 546
N. Y. State Medical Society—Transactions of for 1859—Notice............ 36
Nitric Acid in Remittent Fever........................ 697
Noeggerath and Jacobi—Contribution to Midwifery, etc.................. 355
Notes and Sketches on Diseases of the Tropics........................ 588
Notice of Dixon on Diseases of the Eye........................
Notices (Objectionable Advertisement, etc.)........................ 384

Notice of Proceedings of Pharmaceutical Association .................... 799
Notice of Storer on Criminal Abortions in America.... .................... 805
Notice of Toynbee on Diseases of the Ear ............................. 803
Notice of Wood's Introductory Lectures and Addresses.............. .... 804
Opium an Antidote to Belladonna.................................... 789
O'Reilly's (Dr. J.) Prizes............................................ 736
Organoscope ........................................................... 729
Osteo-Plastic Operations ............... ............................ 726
Our Hospital Articles (Editorial).........................254, 319, 512, 575
Ovariotomy ............. ........................... 628, 795, 872
Paine's Institutes of Medicine .. ................................... 448
Pancreas—Digestion of Albuminoids by, (Corvisart) ............... .... 301
Paralysis of Motion—Treatment of.................................. 782
Parisian Medicine and Surgery—Monthly Review of ........24, 72, 151, 274, 533
Parisian Confrere as Deputy Physician ........ ........................ 64
Peaslee (Dr. E. R.) on Displacements of the Uterus............577, 668, 758, 852
Perchloride of Iron in Diphtheritis.................................. 610
Pharmaceutical Preparations—Report on............................ 865
Philadelphia Hospital ................................................ 319
Physiology of the Circulation ....................321, 585, 449, 513, 757, 830
Physicians, Educate your Patients........................ ............... 312
Physiology of the Circulation—Lectures of Prof. Dalton, 321, 385, 449, 513, 737, 830
Physiological and Chemical Changes in the Body (Dalton) ................ 232
Phymosis with Adhesion of the Prepuce, by Wm. H. Butler, M.D. ..... ... 421
Physicians' Hand-Book of Practice ....... ............................. 508
Pig—Intestines of, after being fed with Typhoid Dejections................ 39
Placenta—New Function of....... ..................................... 114
Pneumonia—Treatment of ............................................. 794
Podophyllin and Leptandrin.......................................... 792
Potash—Chlorate of, in Acute Rheumatism ............................ 625
Proceedings of Pharmaceutical Association—Notice of.... ................ 799
Prolapse of the Funis—Treatment of ............................616, 792
Psoas Abscess—Case of Appearing at Crural Ring, by J. B. Trembley, M.D. 69
Puerperal Convulsions................................................. 619
Pyæmia ............. .................................................. 817
Quinine in Uterine Hæmorrhage ....................................... 786
Raw Meat as a Remedial Agent........................................ 785
Rankin's Abstract—Notice of ......................................... 806
Remarkable Obstetrical Case ......................................... 119
Report on Pharmaceutical Preparations................................ 865
Rochester Knockings Again ........................................... 186
Rogers (Dr. W. C.) on Resuscitation of Children Born Still................ 771
Sayre (Dr. L. H.) on Morbus Coxarius.................................. 712
Scarlatina...............................................709, 711, 787
Smith (Dr. E. P.)—Case of Vesico-Vaginal Fistula....................... 148
Sore Nipples.......................................................... 879
Specialties in Medicine and the Paris Faculty .......................... 733
Spinal Cord—Transmisssion of Sensation by Ant. Colum. (Nonah)—Trans. by L. Damerinville.............................................. 34

Spleen—Human—Extirpation of (Martini)........................ 566
Sternum, Congenital Fissure of—Remarks on Case of E. A. Groux, by Austin Flint, M.D. ........................................ 1
Stomach—New Mode of Treating Chronic Inflammation of (Alex. Fleming).. 373
Stomatitis of Nursing Women........................................621, 702, 880
Strychnia in Seminal Emissions ........................................ 703
Subcutaneous Injection ........................................ 878
Sugar in the Urine—Examination of ........................................ 605
Suicides in Sweden ........................................ 733
Summary of Medical Journalism........................................630, 712, 784, 872
Swallowing Teeth ........................................ 794
Tannin in Affections of the Uterus ........................................ 735
Tanner on Diseases of Infancy and Childhood ........................................ 299
Tape-Worm Dependent upon Use of Raw Meat........................................ 179
Taylor on Poisons ........................................ 300
Taylor (Dr. C. F.)—Paralysis of Motion........................................ 782
Throat Deafness—Cases of (Dr. Lente) ........................................ 370
Tobacco in Rattle-Snake Bites........................................ 701
Tongue Removed by the Ecraseur ........................................ 703
Tonic for Dyspepsia. ........................................ 790
Toynbee on Diseases of the Ear—Notice of........................................ 803
Trachea— Ulceration of, Produced by Canula after Tracheotomy........................................ 122
Transfusion ........................................ 706
Traumatic Diabetes ........................................ 384
Treatise on Fractures and Dislocations—Hamilton's—Review of........................................ 797
Trembley (Dr. J. B.)—Case of Psoas Abscess........................................ 69
Trismus Nascentium Treated with Quinine ........................................ 878
Tumors of Breast—Topical Application for ........................................ 693
Uterine Hæmorrhage........................................ 792
Uterine Polypus Treated by Injection of Perchloride of Iron........................................ 599
Uterus—Case of Rupture of........................................ 124
Uterus—Chronic Inversion of (Ramsbotham)........................................ 565
Valeriniate of Strychnia ........................................ 795
Value of Scientific Services ........................................ 319
Van Arcken (Dr. G.) on Diseases of the Tropics........................................ 588
Variola and Vaccina ........................................ 791
Vesico-Vaginal Fistula ........................................ 697
Vesico-Vaginal Fistula—Case of, by E. P. Smith, M.D. ........................................ 148
Voltaic Narcotism ........................................ 611
Vomiting in Pregnancy........................................ 879
Waggoner (Dr. John S.) on Lumbar Abscess........................................ 768
Water Dressings........................................ 708
Wertzer's Cure for Hernia........................................ 705
Wood (Dr. Geo. B.)—Introductory Lectures and Addresses—Notice of .... 804
Woorara—Effects of, Introduced into the Stomach, by Dr. D. Brainard ..... 110
Woorara in Tetanus........................................ 575
Wounds of the Scalp ........................................ 789
Wound of the Brain........................................ 703
Yellow Fever—New Treatment for........................................ 873
Yellow Fever Useful ........................................ 190

☞ Exchanges, Correspondents and Publishers will please send all communications, journals, books for review, and letters relating to the editorial department to AUSTIN FLINT, JR., EDITOR OF THE NEW YORK REVIEW AND BUFFALO MEDICAL JOURNAL, NEW YORK CITY. All remittances, commissions and letters referring to the advertising and business department will be sent to A. I. MATTHEWS, PUBLISHER, BUFFALO, N. Y.

We hope our exchanges and correspondents will make this change so that our journals, etc., may be received without unnecessary delay. The business will still be transacted by Mr. A. I. Matthews, who will receive all remittances, and contract for advertisements. Owing to the changes which have been made, the present number is delayed for a few days, but hereafter each number will appear on the first of the month as heretofore.

